# Super active surveillance for low-risk prostate cancer | *Opinion: No*

**DOI:** 10.1590/S1677-5538.IBJU.2019.02.03

**Published:** 2019-04-01

**Authors:** Saum Ghodoussipour, Amir Lebastchi, Peter Pinto, Andre Berger

**Affiliations:** 1 Department of Urology, University of Southern California, Los Angeles, California, USA; 2 National Cancer Institute - NCI, Bethesda, Maryland, USA

**Keywords:** Prostatic Neoplasms, Risk Reduction Behavior, Watchful Waiting, Therapeutics

## INTRODUCTION

Prostate cancer (PC) is diagnosed in over 170,000 men in the United States each year. While this makes PC one of the most common solid malignancies in men, a significant majority will not die from PC but from other unrelated causes (1). In fact, almost half of men with screening detected and localized PC are considered candidates for deferred treatment (2). In an effort to decrease the morbidity associated with overtreatment, guideline panels now recommend active surveillance (AS) for those with low risk (LR) disease (3-5).

The use of AS has been steadily increasing and is supported by large cohort studies showing 98-100% PC specific survival (6, 7). While the recommended follow-up for AS varies, safety is predicated on close surveillance with predefined thresholds for treatment based on identification of progression to life threatening but still curable disease. In the largest published AS cohort of 993 men with median follow-up of 6.4 years, 10-year cancer specific survival (CSS) was 98.1%. However, 27% of these patients ultimately underwent surgery for indications ranging from prostate specific antigen (PSA) progression, biopsy Gleason score progression or patient preference. While this cohort included mostly younger men with low risk disease (Age <70, cT1/T2a disease, PSA <10ng/ml), they also included patients older than 70 with Gleason 7 or lower disease, such that 20% had intermediate risk (IR) disease (6). A separate analysis of this cohort by Musunuru et al. showed that while only 3% of patients developed metastases, metastasis free survival (MFS) was significantly lower in the IR as compared to the LR group (84% vs 95%, p=0.001) (8). Another separate cohort analysis by Yamamoto et al. showed a significantly higher risk of 15-year PC mortality (PCM) for higher Gleason score disease (HR of 4.0 for Gleason 3+4 vs 3+3 and HR 10.5 for Gleason 4+3 vs 3+3) (9). In the PROTECT trial, which included men with localized PC, 1643 were randomized to AS (n=545) or definitive treatment with radical prostatectomy (RP) (n=553) or radiation therapy (RT) (n=545). There was no difference in PCM amongst the 3 groups (p=0.48), but of the 17 patients who did die, 8 were in the AS group (5/8 with IR disease), 5 in the RP group and 4 in the RT group. The rate of disease progression and development of metastases was significantly higher in the AS group as compared to RP or RT (112 vs. 46 vs. 46 men, respectively, p<0.001) (10).

Despite a certain subset of patients who seem to do worse with AS, concerns with morbidity from definitive treatment have lead experts to recommend a broadening of the indications for AS to include those with IR disease (3, 5, 11, 12). As the indications for AS expand, certain patients may wish to be even more active in their surveillance. In 2018, Bloom et al. proposed the concept of “Super Active Surveillance”, which they defined as focal ablation of an index lesion in order to alleviate concerns of disease progression or ultimate need for definitive treatment (13). While studies have shown the feasibility of ablative techniques, the use of Super AS remains a work-in progress and should be considered an experimental treatment only performed in the hands of well-experienced providers, ideally as part of an investigational study. Herein, we explore the rational behind Super AS and address the lingering but significant questions that require answering before adoption of this as a mainstream approach.

### Multiparametric MRI and the changing paradigm in prostate cancer diagnosis

The diagnosis of PC has classically been via systematic ultrasound guided biopsy. However, this method under stages 30% of men with PC (14-17). This is thought to be due to under sampling or poor visualization of hard to reach areas such as the apex or anterior zones. Multiparametric magnetic resonance imaging (mpMRI) has emerged as an important diagnostic tool in PC as it allows more accurate sampling of the prostate so that clinicians will identify more clinically meaningful PC while avoiding overtreatment of clinically insignificant disease (18, 19). The enhanced ability of mpMRI to detect significant disease comes from fusion biopsy techniques where direct targeting of suspicious lesions not seen on ultrasound may be performed (20, 21). The use of mpMRI is now recommended by guideline panels to confirm eligibility for AS and to rule out significant cancers (3-5).

While MRI targeted fusion biopsy is now the preferred approach, some have even proposed a role for mpMRI to replace biopsy in those on AS (22-25). Data supporting the practice of MRI as a replacement for repeat biopsy come from single centers who are well experienced, so interpretation should come with caution especially as mpMRI may miss up to 15% of clinically significant tumors (26). Margel et al. found an 83% positive and 81% negative predictive value for mpMRI in reclassifying patients who no longer met criteria for AS (22). A recent study by Panebianco et al., included 1,255 men with negative mpMRI who were treated at a tertiary referral center. A prior negative biopsy had been performed in 596 men and 659 were biopsy naive. These men were followed for a minimum of 2 years and freedom from any PC was 94% overall. At 4 years, the freedom from any grade prostate cancer was 84% for those who were biopsy naïve and 96% in those with a prior negative biopsy (27).

Thus, mpMRI clearly enhances diagnosis but systematic biopsy is still needed to prevent a missed cancer diagnosis in those at risk but with negative mpMRI. Certainly, larger prospective multi-institutional studies are needed in those with negative imaging. In those with positive imaging however, fusion biopsy not only improves detection but also may serve as a useful guide for minimally invasive image-guided treatment (13).

### Focal ablation: feasible but safe?

The acceptance of image-guided diagnosis in PC has spawned the era of image-guided treatment, also known as focal ablation. Focal ablation is defined as the specific targeting and ablation of the malignant portion of the prostate while leaving benign tissues intact. Methods of ablation vary and include cryotherapy, high intensity focused ultrasound (HIFU), radiofrequency ablation, laser ablation, irreversible electroporation, microwave ablation, photodynamic therapy and water vapor therapy. Feasibility of each treatment has been shown in whole gland and partial gland ablation, but level one evidence is lacking as studies consist mostly of single center cohorts without long-term follow-up (13).

Focal ablation is based on the hypothesis that an index lesion, the biggest and highest-grade Gleason lesion, drives cancer related outcomes (28-31). However, PC is known to be a multifocal disease with unilateral disease occurring in only 20-30% of cases (30-32). Just as negative mpMRI may miss disease, focal ablation has the potential to miss cancer and risk progression. Before focal ablation or “Super AS” can be considered a safe option for patients otherwise considering AS, the ideal candidate, follow-up and definition of treatment failure must be defined.

The ideal patient for focal ablation is still debated without consensus or long-term data. Gill et al. showed the safety of focal ablation in men with low risk PC (Gleason score 6, cT2a, PSA ≤10). They compared AS or focal ablation with targeted photodynamic therapy in 413 men and found a lower conversion to radical therapy in the ablation group compared to the AS group (24% vs. 53% at 4 years, HR 0.31, 95% CI 0.21-0.45). Cancer progression rates were also lower in the ablation group (HR 0.42, 95% CI 0.29-0.59) (33). European Association of Urology (EAU) has put forth a position statement on focal ablation acknowledging that most reports have included men with Gleason 6 disease, but that those with IR risk disease (Gleason ≤4+3) may be considered just as they are for AS (34).

Gland and tumor specific variables must be considered as well. For example, the ideal gland size for HIFU is 40g and must be without calcifications that may interrupt ultrasound wave transmission (35). In a study by Truesdale et that evaluated patient selection criteria for unilateral cryoablation, they found that pre-treatment PSA, Gleason score, number of cores positive and total tumor length were associated with biochemical and pathologic disease progression (36).

Appropriate follow-up for those on Super AS must be defined such that treatment failure requiring conversion to more radical therapies can be reliably predicted. Biochemical recurrence (BCR) is a primary endpoint in predicting treatment failure after RP or RT, but no universal criteria for BCR exist after focal ablation. While residual disease may exist after focal ablation and lead to progression, PSA is not a good predictor of this risk (37). Viable and benign prostate tissue will continue to produce PSA. Moreover, PSA kinetics in a partially ablated gland differ from those following whole gland ablation, RP or RT (38). Results of repeat biopsy due to PSA based changes are highly variable as studies have found residual disease in 8-45% of cases (36, 39, 40). Routine biopsy performed one year after ablation similarly shows variable rates of residual disease with disease in 0-26% of cases (37, 38, 41, 42). Some have proposed a MRI based method of detecting recurrent disease after focal ablation (43) but inability to differentiate disease vs treatment and presence of disease despite negative MRI remain concerns (37).

The decision to end AS and proceed to more aggressive treatments currently depends on deterioration of inclusion criteria and not just worsening of mpMRI features or development of new lesions on their own (5). Given the considerable uncertainties in follow up after focal ablation, EAU recommends that patients should be treated only within the context of a clinical trial using predefined criteria (34).

## CONCLUSIONS

Paradigm shifts are underway in the management of prostate cancer. AS is a safe and recommended option for patients with LR disease and a select group with IR disease. Concerns over disease progression and eventual need for definitive treatment have driven patient interest in alternative options to AS that still avoid the morbidity or surgery of radiation.

The use of mpMRI and fusion biopsy has greatly enhanced urologists ability to diagnose prostate cancer and to determine patients’ candidacy for AS. Improved imaging has also allowed identification of an index lesion that may or may not be the driver of oncologic outcomes. While focal ablation of these lesions is technically feasible, we are in need of larger, prospective studies with adequate follow up in order to determine true oncologic outcomes. Significant questions remain regarding the appropriate candidate for Super AS, follow up as well as triggers for conversion to more aggressive therapy.

While patient driven excitement may influence urologists to pursue Super AS, its use should be reserved for high volume centers with a dedicated focal ablation team under a strict investigational protocol. While an exciting option for consideration, Super AS should be considered an experimental option not yet ready for prime time.
